# THE COMPLIANCE OF PSYCHIATRIC PATIENTS WITH HYPERTENSION TO MEDICATION AND FOLLOW-UP DURING THE COVID-19 PANDEMIC

**DOI:** 10.1192/j.eurpsy.2023.1683

**Published:** 2023-07-19

**Authors:** M. E. Anagnostaki, I. Anagnostaki, G. Papaspiropoulou

**Affiliations:** Internal Medicine Dpt, Psychiatric Hospital of Attika, Athens, Greece

## Abstract

**Introduction:**

The current SARS Covid-19 pandemic has negatively affected primary care and health system services provided to chronically ill patients, such as patients with Diabetes Mellitus, Dyslipidemia and Hypertension.

**Objectives:**

The recording of the number of unfulfilled scheduled visits of Psychiatric patients who are monitored in the Pathological Outpatient Clinic of Arterial Hypertension at the Psychiatric Hospital of Attica due to non-attendance, before the pandemic (9/2018 -2/2020) and during its evolution (3/2020-2 /2022).

**Methods:**

The study was done retrospectively and included 1515 patients with a Psychiatric history and concomitant Hypertension who were examined at Outpatient Clinics during the aforementioned time intervals. The rates of missed scheduled visits, as well as discontinuation of treatment without a doctor’s indication were compared and the statistical method used was χ2 with a significance level of p<0.05.

**Results:**

The percentage of scheduled appointments that did not take place due to patient no-shows during the pre-pandemic period was 22% (550/2500). During the pandemic the non-attendance rate increased to 36% (1132/3145). The increase in the rate of missed appointments was statistically significant with p<0.001. Correspondingly, the difference in recorded discontinuation of treatment was statistically significant, 26% (523/2011) versus 33% 743/2252).

**Conclusions:**

Στην παρούσα μελέτη διαπιστώνεται στατιστικά σημαντική αύξηση της μη προσέλευσης Ψυχιατρικών ασθενών στα Παθολογικά Εξωτερικά Ιατρεία τη περίοδο της πανδημίας. Επίσης το πρόβλημα της μη συμμόρφωσης στη συνιστώμενη αγωγή διογκώθηκε καθώς καταγράφηκε μεγαλύτερο ποσοστό μη συνιστώμενης διακοπής αγωγής για την Αρτηριακή Υπέρταση .

**Disclosure of Interest:**

None Declared

